# Rapid Colorimetric pH-Responsive Gold Nanocomposite Hydrogels for Sensing Applications

**DOI:** 10.3390/nano12091486

**Published:** 2022-04-27

**Authors:** Ahmed E. Salih, Mohamed Elsherif, Fahad Alam, Matteo Chiesa, Haider Butt

**Affiliations:** 1 Department of Mechanical Engineering, Khalifa University, Abu Dhabi P.O. Box 127788, United Arab Emirates; mohamed.elsherif@ku.ac.ae (M.E.); fahad.alam@ku.ac.ae (F.A.); matteo.chiesa@ku.ac.ae (M.C.); 2 Department of Physics and Technology, UiT The Arctic University of Norway, 9010 Tromsø, Norway

**Keywords:** nanocomposites, Biosensors, pH sensors, colorimetric sensing, optical sensors

## Abstract

Surface functionalization of metallic nanoparticles (NPs) with external groups can be engineered to fabricate sensors that are responsive to various stimuli like temperature, pH, and numerous ions. Herein, we report the synthesis of gold nanoparticles (GNPs) functionalized with 3-mercaptopropionic acid (GNPs-MPA) and the doping of these nanoparticles into hydrogel materials using the breathing-in/breathing-out (BI-BO) method. MPA has a carboxyl group that becomes protonated and, thus, ionized at a pH below its pK_a_ (4.32); hence, the GNPs-MPA solutions and gels were mostly pH-responsive in the range of 3–5. Optical properties were assessed through ultraviolet-visible (UV-Vis) spectroscopy, namely: transmission and absorption, and the parameters used to quantify the pH changes were the full width at half maximum (FWHM) and position of surface plasmon resonance (SPR). The solutions and gels gradually changed their colors from red to indigo with pH decrementation from 5 to 3, respectively. Furthermore, the solutions’ and doped gels’ highest FWHM sensitivities towards pH variations were 20 nm and 55 nm, respectively, while the SPR’s position sensitivities were 18 nm and 10 nm, respectively. Also, transmission and scanning electron microscopy showed synchronized dispersion and aggregation of NPs with pH change in both solution and gel forms. The gel exhibited excellent repeatability and reversibility properties, and its response time was instantaneous, which makes its deployment as a colorimetric pH-triggered sensor practical. To the best of our knowledge, this is the first study that has incorporated GNPs into hydrogels utilizing the BI-BO method and demonstrated the pH-dependent optical and colorimetric properties of the developed nanocomposites.

## 1. Introduction

Owing to their interesting optical, electronic, and chemical properties, gold nanoparticles (GNPs) have been used extensively in biomedical and chemical related industries [1,2,3,4,5,6]. In a myriad of applications, GNPS undergo surface functionalization, which either enhances the intrinsic properties of these nanoparticles (NPs) or introduces new ones [7,8,9,10]. Some of the commonly used functional groups for gold nanoparticles include thiols, carboxylates, and amines. Surface functionalization with such groups allows gold nanoparticles to exhibit physiochemical properties that are responsive to external stimuli, such as pH, temperature, and ions, making them attractive for biosensing and other biological applications [11,12].

Recently, pH-triggered colorimetric changes of functionalized gold colloidal NPs were studied and utilized in numerous applications [13,14,15,16,17,18,19]. For example, Nam et al. functionalized GNPs with an amide group and used their pH-responsive behavior in photothermal cancer therapy [11]. Thiols have widely been used as pH-triggered molecules and attached to GNPs. For instance, Ansar et al. fabricated MUA-functionalized gold nanoparticles and applied their pH-triggered aggregation and re-dispersion properties in catalysis [7]. Authors showed that the GNPs aggregated at pH 4.1 due to MUA protonation, resulting in reduced surface charge. Yet at pH 8.7, MUA ionization occurred and phase transfer into an immiscible organic phase was induced. Further, optimum MUA surface coverage, which ensures adequate colloidal stability and catalytic activity was reported at 90%. The significance of the developed catalyst was apparent in its ability to show excellent recovery and reusability at different pH regimes. Fan et al. [7] developed responsive nanovesicles by self-assembly of GNPs with mercaptobenzonic acid (4-MBA) and oleylamine (OL) [20]. Self-assembled nanovesicles dissociated into monodisperse NPs at alkaline conditions (pH 11.7), yet those NPs reassembled into nanovesicles when placed in an acidic solution (pH 4.8). Similar to previous studies, this reversible behavior was due to the deprotonation and protonation of 4-MBA. Reversible changes of the NPs were confirmed through captured transmission electron microscopy (TEM) images and measured surface plasmon resonance (SPR) position of the assembled and disassembled nanovesicles. Authors also tested the adequacy of the developed pH-responsive gold nanovesicles for drug release applications by loading the NPs with Rhodamine B (RhB), which has a fluorescence emission at around 575 nm, and monitoring the release of RhB as the pH was varied. Results showed that the fluorescence intensity increased gradually with pH incrementation until all RhB molecules were released entirely, upon which the fluorescence intensity became stagnant. Thus, these pH-responsive gold nanovesicles are good candidates for biosensing and controlled drug release. Moreover, Shiraishi et al. [20] grafted 3-mercaptopropionic acid (MPA) into gold nanoparticles (GNPs-MPA) and studied their optical-colorimetric response at different pH values [21].The developed GNPs-MPA exhibited a reversible colorimetric change between red and purple in basic and acidic conditions, respectively. The authors attributed the resulting change in color to the degree of NPs’ dispersion, which they confirmed through TEM micrographs of the GNP at different pHs. Although the pK_a_ of MPA is 4.34, authors did not report the pH at which their developed gold nanoparticles became precipitated and heavily aggregated [21].

The aforementioned studies examined extensively multiple pH-responsive molecules, added them to GNPs, and utilized them for different applications. However, the optical and colorimetric behavior of pH-responsive GNPs integrated into hydrogels has yet to be thoroughly explored. Hydrogels have been utilized to fabricate smart sensing optical fibers, contact lenses, and skin patches [22,23,24,25,26,27,28]. More recently, gold and silver nanoparticles were introduced in contact lenses as color filtering devices for color blind patients [29,30]. The latter highlights few of the contemporary research done in which nanoparticles were integrated with hydrogels for biomedical applications. Hence, successful incorporation of colorimetric pH-responsive GNPs into hydrogel materials could pave the way for the development of a wide range of nano-sensing devices.

In this study, pH-responsive GNPs-MPA were synthesized and incorporated into hydrogels via the breathing-in/breathing-out (BI-BO) method, which has been previously utilized to integrate NPs into hydrogel materials [31,32,33,34]. Optical properties of the developed GNPs-MPA solutions were characterized through ultraviolet-visible (UV-Vis) spectroscopy at different pH values. The morphology of the colloidal nanoparticles at distinct pHs was examined through the TEM. After using the BI-BO method, the developed nanocomposites were characterized for their optical and morphological properties at different pHs. The colorimetric red, green, and blue (RGB) data from each hydrogel was analyzed as the pH was altered. The optical properties used to quantify the sensor’s response were the SPR position and full width at half maximum (FWHM). The repeatability, reversibility, sensitivity, and response time capabilities of the developed nanocomposite sensor were also reported and analyzed.

## 2. Materials and Methods

### 2.1. Materials

Chloroauric acid (HAuCl_4_, 99.99% trace metals basis), 3-mercaptopropionic acid (MPA, ≥99.0%), trisodium citrate dihydrate (Na_3_C_6_H_5_O_7_·2H_2_O, ≥99.0%), sodium hydroxide (NaOH, ≥98.0%), acetone (C_3_H_6_O, ≥99.5%), and hydrochloric acid (HCl, 37%) were purchased from Sigma Aldrich (St. Louis, MO, USA) and used as is without further purification. Acuvue Oasys 1-Day with HydraLuxe contact lenses were obtained from Acuvue, Johnson & Johnson^®^, New Brunswick, NJ, USA.

### 2.2. Preparation and Fabrication of Gold Nanoparticles Protected by MPA

Colloidal GNPs-MPA were synthesized utilizing the well-known Turkevich citrate-reduction method [35,36]. 17 mg of HAuCl_4_ were dissolved in 100 mL of deionized water (DI) and heated to boiling point. Prior to that, 0.1 M sodium 3-mercaptopropionate (MPA-Na) was prepared by mixing 175 µL of MPA with 80 mg of NaOH in 20 mL of DI water. 200 µL of the latter was added to 3.87 mL of 1% trisodium citrate dihydrate. When the precursor solution reached boiling, the aforementioned mixture was added and stirred for 3 min, upon which the color turned red. Then, the mixture was left to stir for 30 min at room temperature and was subsequently stored at 4 °C.

### 2.3. Breathing of GNPs-MPA into Hydrogel Matrix

**Figure 1:**
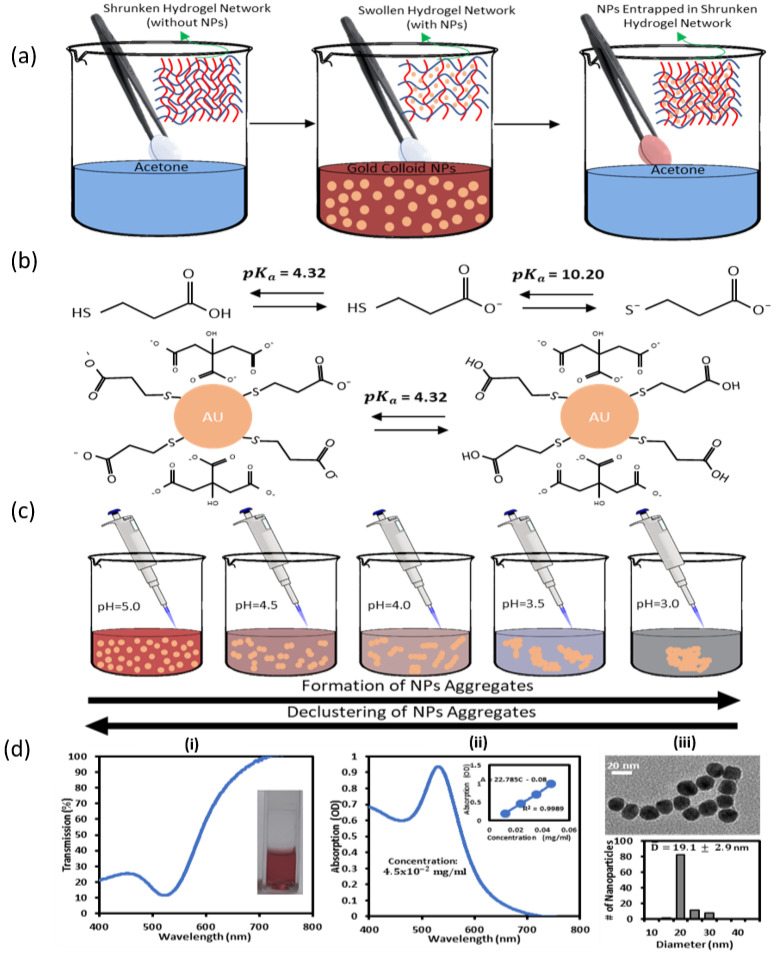
Gold-MPA nanocomposite hydrogel’s synthesis and mechanism of formation. (**a**) Breathing process utilized for loading the nanoparticles into the hydrogel matrix. Protonation of (**b**) MPA (at pK_a_ = 4.32 and pK_a_ = 10.20) and gold nanoparticles protected by MPA (at pK_a_ = 4.32). (**c**) Illustration of pH-dependent reversible colorimetric change of gold colloidal NPs. (**d**) Synthesized gold nanoparticles protected by MPA. (**i**) Transmission and (**ii**) absorption spectra. Inset shows the calibration curve used to determine the concentration of the NPs. (**iii**) TEM of the synthesized gold nanoparticles (top) along with their corresponding size distribution (bottom).

Colloidal GNPs-MPA were incorporated into the hydrogels utilizing the BI-BO method [31,32,33]. Contact lenses were used as hydrogel materials and were purchased from Johnson & Johnson^®^. The hydrogels were placed in 5 mL of acetone for 2 min, after which the gels became shrunken. Then, they were kept in 5 mL of the synthesized GNPs-MPA colloid for 5 min; gels were swollen at this stage. Subsequently, when the gels were placed again in acetone, the NPs remained inside their network while water was expelled. This process was repeated to show the effect of the number of cycles on the gel’s optical properties. Gels were washed with DI water to remove unadsorbed NPs. The fabrication scheme is demonstrated in Figure 1a.

### 2.4. Preparations and Testing of GNPs-MPA Solutions and GELs at Different pHs

GNPs-MPA colloids at distinct pHs were prepared by adding 1 mL of the colloid to 1 mL of DI water, which was adjusted using 0.1 M HCl/NaOH to reach the desired pH. Although the pH of the prepared colloids ranged from strongly acidic to basic (2 to 8 respectively), most of these solutions were designed around the pK_a_ of MPA (4.34). Moreover, two GNPs-MPA gels with different concentrations (number of BI-BO cycles) were tested in five different pH solutions. The pH of the solutions was measured using Atlas Scientific’s Lab Grade pH Probe.

### 2.5. Characterization of pH-Responsive GNPs-MPA Solutions and Gels

Optical and morphological characterizations were primarily done to assess the pH responsive behavior of the developed GNPs-MPA solutions and doped gels. The transmission and absorption spectra of the GNPs-MPA solutions were obtained using USB 4000+ spectrophotometer (Ocean Optics, Douglas Avenue, Dunedin, FL, USA) with operation range of 200–1100 nm. 1 mL of each solution was deposited into a cuvette, and the transmission and absorption spectra were recorded using OceanView software. Tecnai TEM 200 kV was used to image the NPs’ morphology at different pHs. A copper mesh grid from Agar Scientific was dipped in 1 mL of the GNPs-MPA solution for 1 min and was then dried in vacuum oven. Size and distribution histograms of the micrographs were done and analyzed using 1.50d ImageJ software.

Furthermore, the transmission and absorption spectra of the doped nanocomposite gels were recorded after 3, 6, 9, 12, and 15 BI-BO cycles. Leakage of the nanoparticles from the gels was assessed over a one-month period and is provided in the supplementary materials. Also, optical spectra of two doped gels that have varying NPs’ concentration were measured upon immersing them in five different pH solutions. Then, the colorimetric response (RGB values) of the gels was processed through ImageJ.

Scanning electron microscopy (SEM) images of the doped gel’s cross-section were done to examine the morphology of the NPs inside the gel; FEI Nova NanoSEM 650, which has an electron beam resolution of 0.8 nm, was used in this study. Initially, the gel was equally sheared, and one part was placed in a basic solution while the other was placed in an acidic solution. Both gels were dried in vacuum oven and coated with 8 nm layer of Gold/Palladium to avoid the charge-up effect.

## 3. Results and Discussion

Morphology and size distribution of the synthesized GNPs-MPA are shown in Figure 1d(iii). NPs were spherical in shape, and their average diameter was 19.1 ± 2.9 nm. Standard deviations of the NPs’ diameter showed that their size was homogenous. Transmission and absorption spectra of the synthesized GNPs-MPA are shown in Figure 1d(i,ii); the SPR occurred at 522 while the FWHM was 56 nm. The narrow bandwidth confirms the assertions made from the TEM images regarding the NPs’ homogenous size distribution.

**Figure 2:**
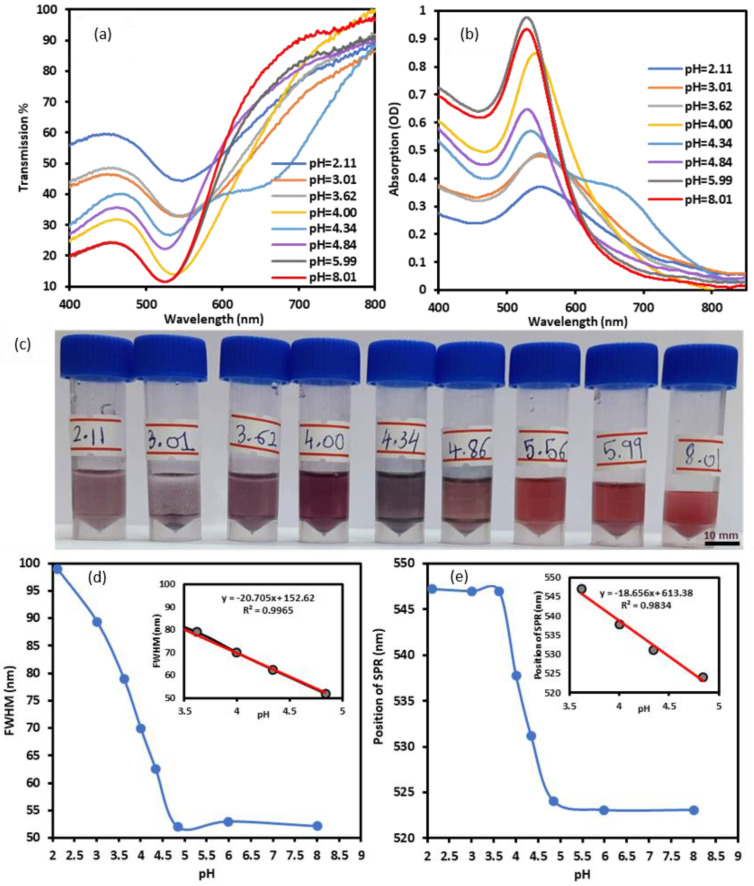
Optical characterization of GNPs-MPA solutions at different pHs ranging from 2 to 8: (**a**) Transmission and (**b**) Absorption spectra of the solutions at distinct pHs. (**c**) Images of the solutions. Extracted information from the optical spectra: (**d**) FWHM and (**e**) Position of SPR as a function of pH. Inset shows the calibration curves in the NPs’ pH-responsive region.

The transmission and absorption spectra of the GNPs-MPA solutions at different pHs were recorded and are shown in Figure 2a,b, and their corresponding images are provided in Figure 2c. Colorimetric changes of the GNPs-MPA solutions were significantly pH-dependent, where the solutions changed color from dark purple to dark red as the pH increased from 2.1 to 8.0. GNPs-MPA solutions with pH 2 and 3 showed that the nanoparticles were aggregated, precipitated, and settled completely whereas NPs with pH values above 5 were reddish in color indicating their even dispersion. Furthermore, the sensitivity of the optical parameters towards pH was deduced from the slope of the calibration curve in Figure 2d,e. Figure 2d illustrates the inverse relationship between the solution’s pH and the absorption’s FWHM, particularly in the pH region less than 5. FWHM sensitivity towards pH was 20.7 nm per pH unit. Likewise, position of the SPR varied between pH 3 and pH 5, and the calculated sensitivity was 18.6 nm per pH unit. The GNPs-MPA’s SPR position was not pH-responsive beyond pH 5. The aforementioned behavior can be explained by examining the functional groups of MPA and the GNPs-MPA. MPA has two functional groups, carboxyl and thiol, which have pK_a_ values of 4.32 and 10.20, respectively. Covalent bonds form between the thiol group and the gold nanoparticles, leaving carboxyl as the only functional group (Figure 1b). Therefore, the high sensitivity of these solutions around pH 3 to pH 5 was expected as the pK_a_ of the carboxyl group is 4.32; GNPs become protonated below that pK_a_. Further, the peak absorption intensity of the GNPs-MPA also diminished as the pH dropped. The absorption intensity at SPR increased by almost 0.6 OD units with the pH incrementation from 2 to 8. This is expected as the pH drop below pK_a_ of MPA causes more NPs agglomeration, which in turn reduces the number of free conduction electrons that can contribute to the surface plasmon. To confirm this, the aggregation/dispersion behavior of the NPs at multiple pHs below 5 was monitored through TEM imaging.

**Figure 3:**
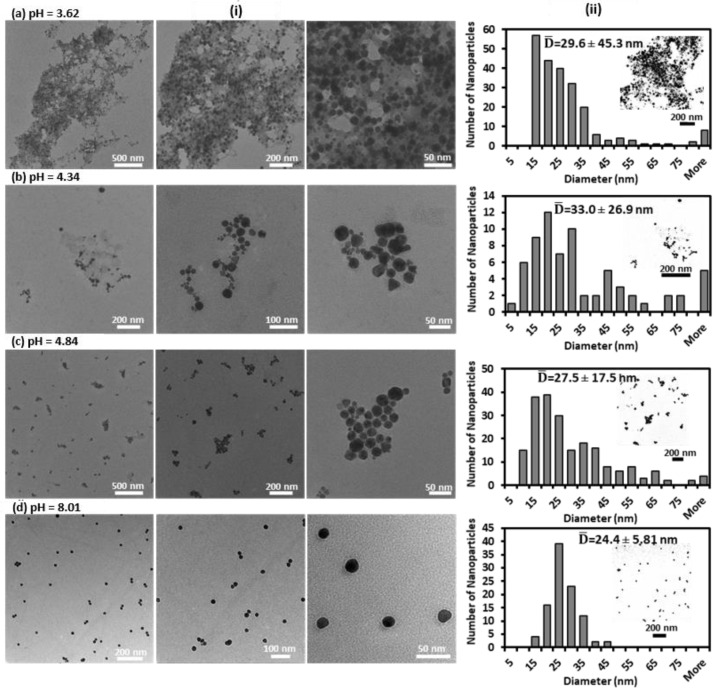
Imaging of the pH-responsive GNPs-MPA solutions: (**i**) TEM micrographs at different scales. (**ii**) Size distribution histograms for pH: (**a**) 3.62, (**b**) 4.34, (**c**) 4.84, and (**d**) 8.01. Inset shows few of the images used for size analysis in ImageJ.

TEM images of the GNPs-MPA solutions at different pHs and their corresponding size distribution are shown in Figure 3, respectively. The NPs were heavily aggregated at strongly acidic conditions, and their dispersion improved as the pH increased. For instance, at pH 3.62, one can note that most NPs were aggregated with few nonclustered nanoparticles, and their size ranged from 11 nm to 511 nm. At pH 8.01, the NPs were evenly dispersed with no signs of big formed clusters, and their measured diameters were in the range of 5 nm to 40 nm. When the solution’s pH decreased below 5, assemblies started forming, and those assemblies became denser as the pH got lower. The average size and morphology of the parent nanoparticles were not altered significantly. The latter remained circular, and the former only varied by less than 10 nm.

However, the polydispersity of the NPs was strongly pH-dependent, which was relayed through the standard deviation of the NPs’ size. As the pH increased from 3.62 to 8.01, the standard deviation of the GNPS-MPA’s diameter reduced from 45.3 nm to 5.8 nm. The latter indicates the NPs became more monodisperse, which can visually be confirmed through the respective TEM images. This behavior of the GNPs-MPA at varying pH conditions is governed by their interparticle interactions, namely: attractive and repulsive forces. At pH values above 5, the repulsive Coulombic forces among the carboxylate groups dominate and cause the NPs to disperse evenly. At pH less than 5, the carboxylic acid group becomes unionized; thus, aggregation occurs as a result of the van der Waals attractive forces [21]. The synchronized alteration of NPs’ dispersion to aggregation state with pH change caused the illustrated change in the solutions’ color from red to purple (Figure 2c). The aforementioned has also been reported with other mercapto compounds, like MUA, which are commonly utilized as GNPs stabilizers [7,37]. It is worth noting that in the absence of MPA citrate-reduced GNP solutions were unresponsive to all pH changes (Figure S1). When the unmodified GNPs solution’s pH was varied, only their absorbance shifted whereas their FWHM and position of SPR remained unchanged.

**Figure 4:**
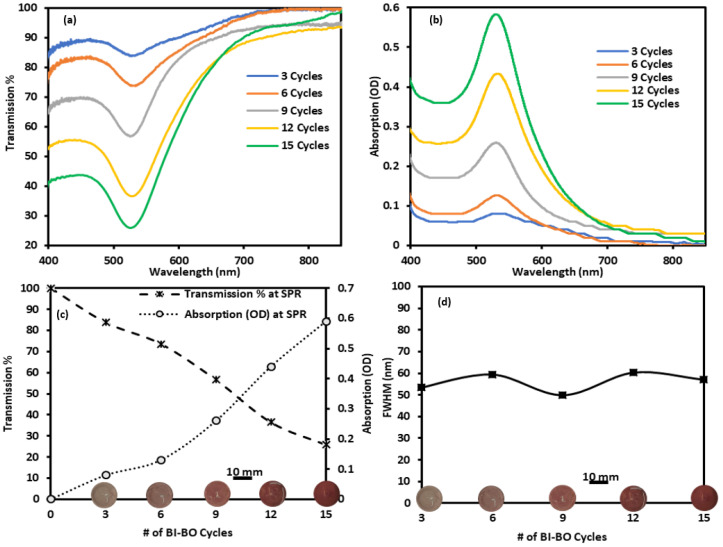
Utilization of BI-BO method to incorporate GNPs-MPA into hydrogels and their corresponding optical properties. (**a**) Transmission and (**b**) absorption spectra of the gels. Variation of the (**c**) transmission percentage, absorption at the surface plasmon, and (**d**) FWHM of the GNPs-MPA gels as a function of the number of BI-BO cycles. Images of the gel at each cycle are shown in the x-axis.

Furthermore, the hydrogels were doped with GNPs-MPA through a simple breathing process described earlier, and the optical spectra over 15 cycles were obtained and are shown in Figure 4. As was shown in previous studies, the higher the number of cycles the higher the overall absorption and, more specifically, the absorption at the SPR [32,33,34,35,36,37,38]. These studies also report that after a certain number of cycles the absorption of the doped gel saturates, suggesting little or no more GNPs can diffuse into the pores of the gel; however, our reported absorption and transmission rate varied consistently and did not plateau over 15 cycles. The transmission at SPR reduced by almost 70% and peak absorption increased by 0.6 OD whereas the FWHM of the absorption peak was unaffected over 15 cycles. These optical changes were also evident from the increased intensity of the gel’s color with increase in the number of BI-BO cycles, as shown in Figure 4c,d. Moreover, leaching of the GNPs from the hydrogel was assessed by placing it in DI water and measuring the optical changes over a one-month period; the recorded transmission spectra over that period is shown in Figure S2. Minimal changes in the spectra of the lenses were noted, suggesting GNPs were stable within the gel.

**Figure 5:**
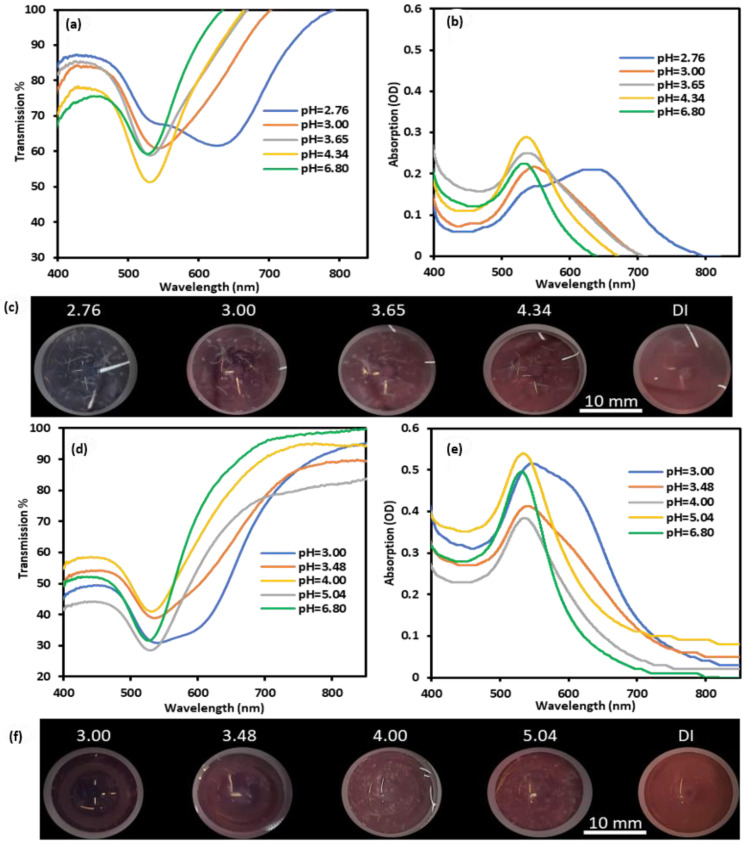
Optical properties of GNPs-MPA doped gels at different pHs: (**a**) Transmission, (**b**) absorption, and (**c**) images of a low concentrated gel (doped with 9 cycles) at different pHs. (**d**) Transmission, (**e**) absorption, and (**f**) images of high concentrated gel (doped with 15 BI-BO cycles) at distinct pH values.

Two gels were doped with 9 and 15 BI-BO cycles and utilized in the pH-responsive study; this was done to show the effect of the NPs’ concentration on the pH-responsiveness of the doped gels. Both gels were placed in five solutions with distinct pH values. Images of the gels and corresponding optical spectra are demonstrated in Figure 5. Similar to the GNPs-MPA solutions, optical and colorimetric properties of the doped gels were also pH-dependent in the range of pH 3–5. Both gels were reddish in color at pH 6.80 (DI water), and as the pH decreased below 5, their colors started changing towards purple/indigo. One can note that for both gels differences were gradual and not abrupt. Nonetheless, below pH 3, the low concentrated gel showed a more apparent change in color. These changes were confirmed through the optical spectra of both gels. The gradual change is evident from the redshift in the transmission/absorption spectra of the GNPs-MPA gels. Below pH 3, the optical spectra of the low concentrated gel showed two transmission dips (absorption peaks) at 545 nm and 629 nm, which indicates high NPs’ aggregation. Similarly, at pH 3, the highly concentrated sample showed an appreciable shift in SPR along with the formation of two transmission dips at 539 nm and 607 nm. Beyond pH 5, the GNPs-MPA doped gels were unresponsive to pH variations, as little or no shift in their absorption spectra was noticed. Also, minimal fluctuations in the transmission at SPR were noted in response to pH changes, which indicates the SPR position and FWHM of the transmission dip were the main optical attributes that were varying.

**Figure 6:**
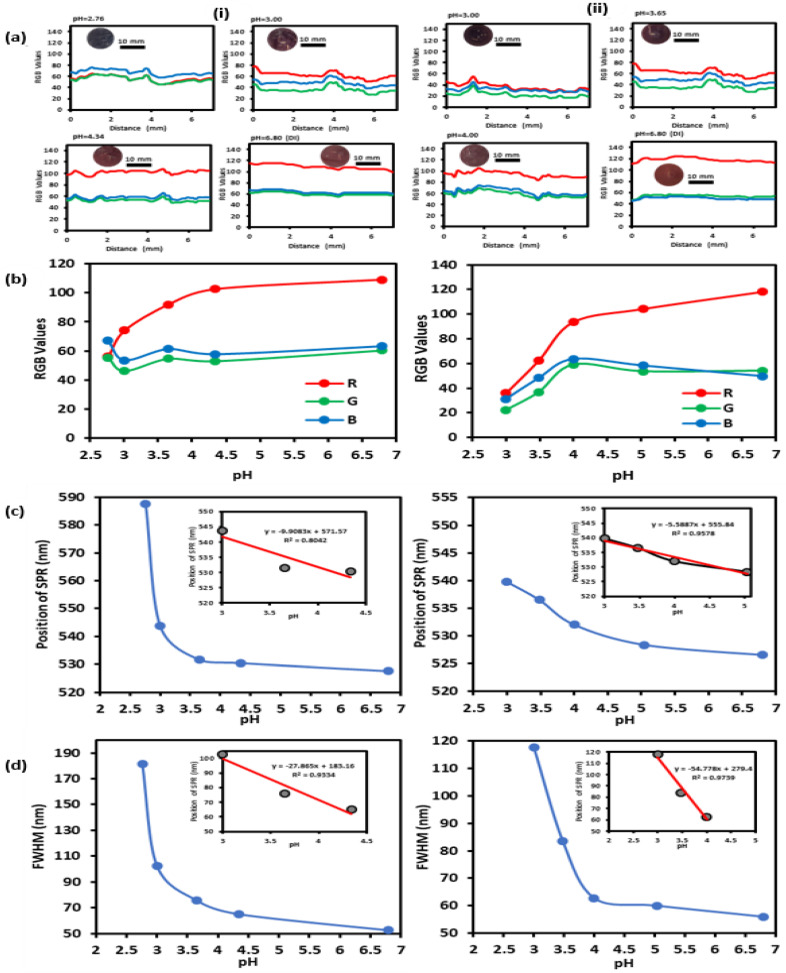
Analysis of the pH-responsiveness of GNPs-MPA doped (**i**) low and (**ii**) high concentrated gels. (**a**) RGB colorimetric analysis of the gel at different pHs using ImageJ. (**b**) RGB of the gel as a function of pH. (**c**) SPR’s position and (**d**) FWHM versus pH. Inset shows the gel’s linear behavior in the pH-responsive range (3–5).

Further, colorimetric analysis was done on 7 mm of each gel at different pH values using the “RGB Profiler” tool in ImageJ, and the RGB data were extracted and are plotted in Figure 6a. Also, the effect of the pH variation on the average RGB values for each gel is shown in Figure 6b. Firstly, the homogeneity of the gel’s color was apparent as major fluctuations in the RGB data plot were negligible. The blue and green curves were overall at a constant value and remained relatively unchanged as the pH was varied. Red curves were generally higher than the blue and green curves except for the low concentrated gel at pH 2.76 for which blue was the highest. This was expected as the gel was violet in color at that pH. Moreover, the general trend was clear: as the pH increased, the red curve shifted upwards indicating the reddish shift in the gel’s color. Hence, the plot of the red curve can be used to relay the real-time value of the pH. Indeed, the red curve of each gel showed a relatively linear behavior as the pH was varied between 3 and 5. The relative change of the red value with pH was 21 units/pH and 39 units/pH for the low and high concentrated gels, respectively.

On the other hand, the position of SPR and FWHM of the doped gels’ transmission spectra were inversely proportional to pH; Figure 6c,d shows the pH-responsive behavior of these optical attributes for both gels. SPR’s position and FWHM of both gels showed the highest variations between pH 3 and 5, yet one of the noticeable discrepancies was evident at pH 2.76, at which the low concentrated gel’s SPR occurred at 587 nm. However, this was reported as an average of the position of the two transmission dips which were at 545 nm and 629 nm and formed as a result of the excessive aggregation. This can be thought of as a saturation limit for the sensor. Hence, the sensor has two saturation limits: (1) Below pH 3, excessive NPs’ aggregation occurs, and color change becomes undistinguishable. (2) Beyond pH 5, MPA becomes deprotonated, and NPs’ color remains unchanged (reddish). As for the sensitivity, the position of SPR varied by almost 10 nm and 6 nm per pH unit for the low and high concentrated gels, respectively. Similarly, the FWHM shifted by 28 nm per pH unit for the low concentrated gel and by twice of that for the high concentrated gel (55 nm/pH unit). The colorimetric and optical variations indicate that the highly concentrated sample was generally more sensitive.

In addition, GNPs were also incorporated into hydrogels without utilizing MPA, and their images and corresponding transmission spectra are shown in Figure S1b. The GNPs doped gels were unresponsive to pH variations as their images showed that they did not change color. The transmission only decreased by 4%, while the SPR and FWHM remained relatively unchanged. These results were similar to the previous findings regarding the pH-responsiveness of the GNPs solutions (without MPA), confirming the idea that functionalization of GNPs with MPA is responsible for their synchronized aggregation/dispersion state.

As for potential applications, these gels can be employed in areas where real-time sensing of strongly acidic fluids might be required. For instance, gastric acid or stomach acid normally has a pH of around 3 [39]. Hypochlorhydria, a condition in which one has low levels of stomach acid due to infections or digestive problems, occurs when the pH of the stomach is between 3 and 5. More severely, if the person’s stomach pH is above 5, he/she is characterized as having achlorhydria, which indicates the complete loss of hydrochloric acid in the stomach [40]. The pH detection limit of the developed gel is within the pH range of the aforementioned conditions. Therefore, continuous monitoring of the stomach acid using this gel can detect and potentially prevent the development of such conditions. The gel can be placed or attached to an optical fiber probe and be used as a continuous pH monitoring device for gastric acid.

**Figure 7:**
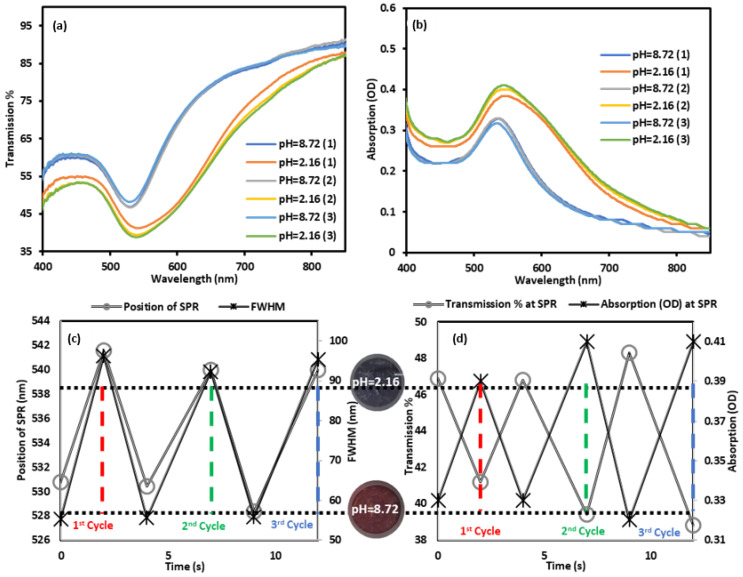
Repeatability and time response of the GNPs-MPA doped gels: (**a**) Transmission and (**b**) absorption of the doped gels over three cycles switching between acidic (pH 2.16) and basic (pH 8.72) conditions (the number next to each curve denotes the number of the cycle). Time response of (**c**) SPR position and FWHM and (**d**) transmission and absorption values at SPR over three cycles. Images of the gel at the two distinct pHs are shown between (**c**,**d**).

Furthermore, the repeatability and time response capabilities of the GNPs-MPA doped gel were assessed by switching it between acidic and basic conditions and recording its optical spectra over three cycles (Figure 7a,b). When the gel was removed from a solution with pH 8.72 and placed in a solution with pH 2.16, the color changed abruptly from red to indigo. SPR’s position redshifted by approximately 14 nm, and FWHM increased by 40 nm. These variations are in-line with the findings presented earlier. Over three cycles, the doped gel showed reversible optical performance (Figure 7c,d). Ranges of variation for the transmissivity, SPR, and FWHM over the three cycles were 2.5%, 2.4 nm, and 4 nm, respectively, which indicate the excellent dispersion/assembly property of the doped gel. Moreover, the colorimetric switch of the doped gel in response to a pH alteration was almost instantaneous as shown in Figure 7c,d. A short video was added to the supplementary data, which confirms instantaneous and reversible change in color as the gel is switched back and forth between basic and acidic solutions. Discrepancies in the time data from the plots (Figure 7c,d) were due to the time difference between placing the sample and taking the measurement.

Moreover, the ability of the gel to be used as a rapid colorimetric/optical switch upon pH change makes it useful for various applications such as drug delivery and biosensing. For instance, drug delivery within the stomach (pH 2–3) or intestine (pH 7) requires a pH-responsive mechanism, and previous studies have focused on pH-triggered electrical, structural, and physical switches [41,42]. Nonetheless, the pH-sensitive doped gel may be used as a colorimetric switch, which is simple yet would not alter the physical properties of the gel like other mechanisms could.

**Figure 8:**
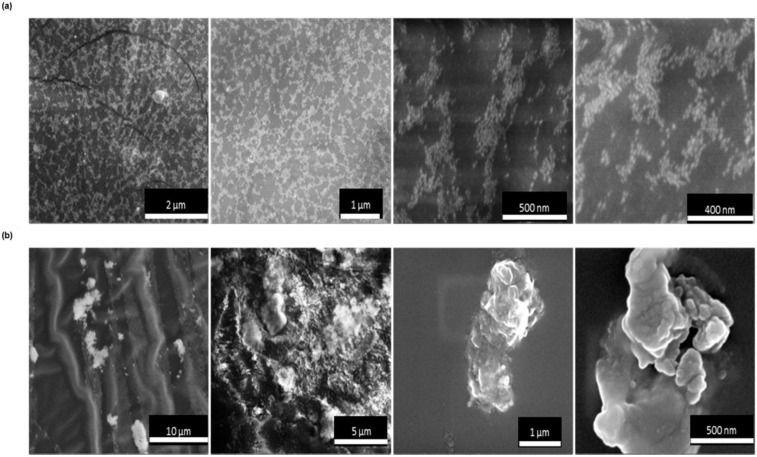
Cross-sectional SEM images, at different magnifications, of the GNPs-MPA doped gel at (**a**) pH 8.72 and (**b**) pH 2.16.

The distribution of the NPs within the pH-responsive doped gel was examined by taking cross-sectional images of the gel after it was placed in both pH solutions (2.16 and 8.72), and the resulting images are shown in Figure 8. Most GNPs at pH 8.72 were evenly dispersed and did not form large assemblies; in fact, their dispersion was similar to the NPs in GNPs-MPA solution at pH 8.01 (Figure 3d). However, at pH 2.16, GNPs were heavily aggregated and formed big clusters. One can observe that the clusters are clearly comprised of numerous NPs. The latter confirms the synchronized dispersion and assembly of GNPs within the gel upon the quick pH variation. These findings were similar to the ones deduced from the TEM images of the GNPs-MPA solutions.

## 4. Conclusions

In this paper, we have synthesized pH-responsive GNPs that were functionalized with MPA (pK_a_ 4.32) and incorporated them into hydrogels. The gel was mostly responsive in the pH range of 3–5. The highest SPR position and FWHM sensitivities towards pH were 10 and 55 nm per pH unit, respectively. Hence, utilization of the FWHM in measuring pH might yield better and more reliable results than using the SPR position. Other optical parameters like the peak absorption intensity were varying non-monotonically with pH, suggesting they cannot be used to infer the pH of the medium. TEM and SEM images showed synchronized dispersion and aggregation of NPs with pH change. The gel displayed high reversibility, repeatability, and instantaneous response time. As it operates in a similar pH range, the gel can be utilized as a continuous pH monitoring device for the gastric acid in the stomach. Also, exploiting its rapid pH-responsive capability, the gel can be used as a colorimetric switch for drug delivery applications. The colorimetric analysis of the gels showed linear relation between the red values and pH; thus, color-detecting applications on smartphones can be developed and employed to relay real-time pH values without the need for optical spectrophotometers. Finally, contact lenses were used as representative hydrogel materials and were successfully doped with GNPs-MPA. These contact lenses could similarly be doped with functionalized pH-responsive GNPs around physiological range (pH 7) to continuously monitor the pH of the eyes’ tears and prevent potential diseases that can occur due to pH fluctuations.

## Data Availability

The data presented in this study are available on request from the corresponding author.

## Supplementary Materials

The following supporting information can be downloaded at: https://www.mdpi.com/article/10.3390/nano12091486/s1, Figure S1: GNPs synthesized without MPA. (i) Images and (ii) absorption and transmission spectra of (a) the solutions and (b) lenses at different pH values.; Figure S2: Transmission test for the MPA-GNPs gel over a one-month period to assess NPs’ leakage.; Figure S3: EDS analysis for the MPA-GNPs gels at pH (a) 2.16 and (b) 8.72.; Figure S4: FTIR analysis of the MPA-GNPs solution.; Video S1: Visual representation of the rapid colorimetric change of the gel as it is switched between pH 2.16 and pH 8.72.
