# Supplementary material for: Rapid Colorimetric pH-Responsive Gold Nanocomposite Hydrogels for Sensing Applications
Source: Nanomaterials (Basel). 2022 Apr 27;12(9):1486. doi: 10.3390/nano12091486 (PMC9101415; doi:10.3390/nano12091486)
Supplement: Supplementary file 1 [file nanomaterials-12-01486-s001.zip › nanomaterials-1677921-supplementary.pdf]

nanomaterials

Supporting Information

Rapid Colorimetric pH-Responsive Gold Nanocomposite Hydrogels for Sensing Applications

Ahmed E. Salih [1,*], Mohamed Elsherif [1], Fahad Alam [1], Matteo Chiesa [1,2] and Haider Butt [1,*]

[1] Department of Mechanical Engineering, Khalifa University, Abu Dhabi P.O. Box 127788, United Arab Emirates; mohamed.elsherif@ku.ac.ae (M.E.); fahad.alam@ku.ac.ae (F.A.); matteo.chiesa@ku.ac.ae (M.C.)
[2] Department of Physics and Technology, UiT The Arctic University of Norway, Tromsø 9010, Norway
* Correspondence: ahmed.salih@ku.ac.ae (A.E.S.); haider.butt@ku.ac.ae (H.B.)

GNPs were synthesized without MPA functionalization, and their pH-responsive properties were studied. As shown in Figure S1a, GNPs were clearly independent of any pH variation even in the pH range of 3-5. The only change was in the absorption intensity, while the FWHM and SPR position remain unchanged. Similarly, a gel doped with unfunctionalized GNPs through the BI-BO method was unsensitive to pH variations although they it was placed in a solution that has pH 3.62. The latter was also evident from the transmission spectrum of the gel.

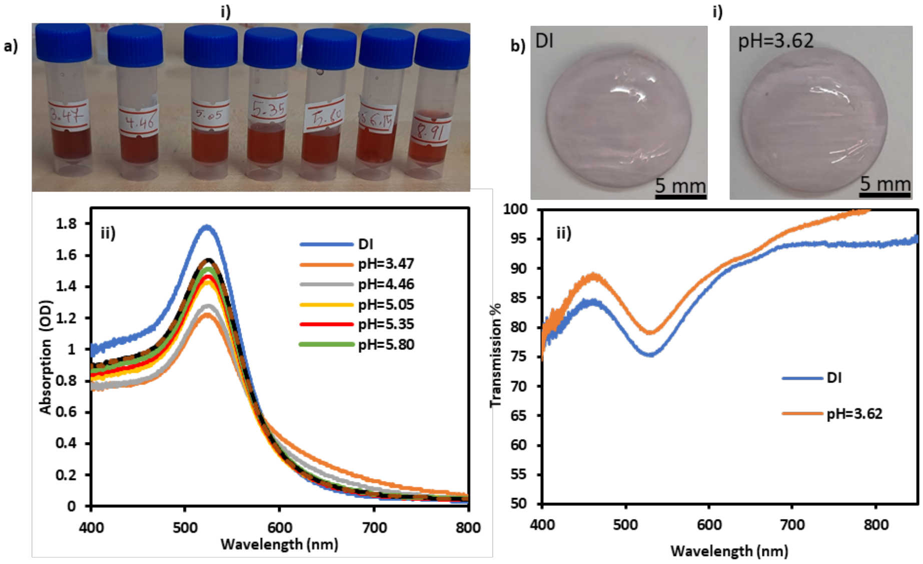

Figure S1. GNPs synthesized without MPA. i) Images and ii) absorption and transmission spectra of a) the solutions and b) lenses at different pH values.

To assess the leakage of the NPs from the gel, its transmission spectra was monitored over a one-month period as it was placed in DI water while continuously changing the water. Since the transmission spectra over the one-month period was almost constant, we can conclude that little or no leakage occurred; also, visually, minimal discrepancies were noted over the test-period.

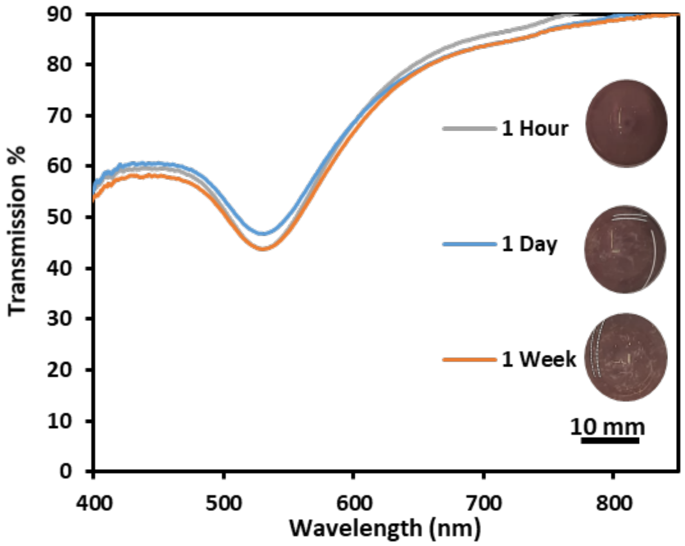

Figure S2. Transmission test for the MPA-GNPs gel over a one-month period to assess NPs' leakage.

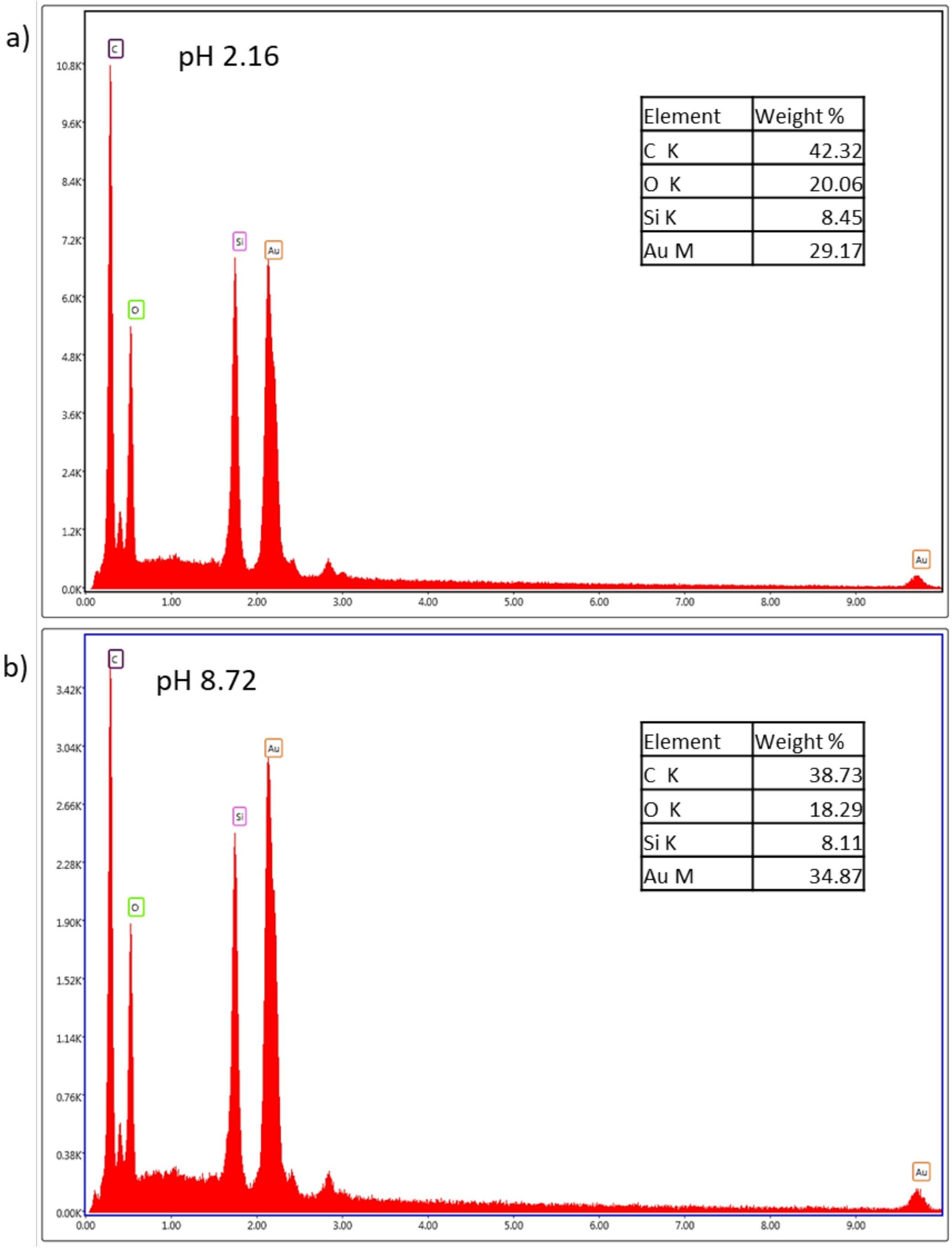

Figure S3. EDS analysis for the MPA-GNPs gels at pH a) 2.16 and b) 8.72.

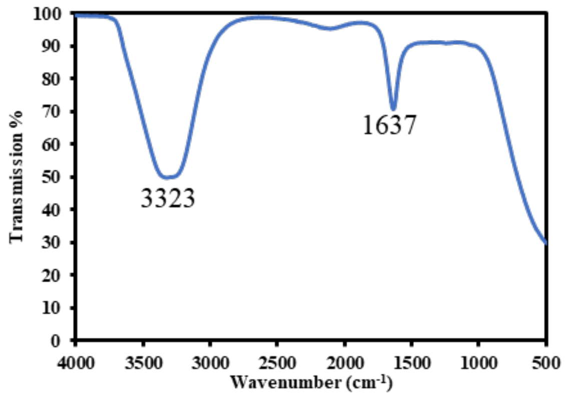

Figure S4. FTIR analysis of the MPA-GNPs solution.
